# Brain imaging biomarkers for the Alzheimer’s Prevention Initiative

**DOI:** 10.1186/1479-5876-10-S2-A28

**Published:** 2012-10-17

**Authors:** Kewei Chen, Adam S Fleisher, Napatkamon Ayutyanont, Jessica B Langbaum, Richard Caselli, Yakeel T Quiroz, Francisco Lopera, Pierre N Tariot, Eric M Reiman

**Affiliations:** 1 Banner Alzheimer’s Institute, Phoenix, Arizona, USA; 2 Med-X Institute, Shanghai Jiao Tong University, Shanghai, China; 3 School of Mathematics and Statistics, Arizona State University, Tempe, Arizona, USA; 4 Department of Neurology, University of California, San Diego, USA; 5 University of Arizona College of Medicine, Phoenix, Arizona, USA; 6 Neurogenomics Division, Translational Genomics Research Institute, Phoenix, Arizona, USA; 7 Center for Memory and Brain, Psychology Department, Boston University, Boston, USA; 8 Grupo de Neurociencias, Universidad de Antioquia, Medellin, Colombia; 9 Department of Neurology, Mayo Clinic in Arizona, USA; 10 Arizona Alzheimer’s Consortium, Phoenix, Arizona, USA

## 

Alzheimer's disease (AD), the most common disabling impairment in older adults, takes an unacceptable toll on patients and families. With the growing number of people living to older ages, it is projected to afflict more than 100 million people around the world by 2050. While there is an urgent need, it takes too many cognitively normal (CN) individuals and too many years to evaluate presymptomatic AD treatments using traditional clinical endpoints.

We and our colleagues have been using imaging techniques to detect the brain changes associated with the predisposition to AD in CN individuals with 2, 1 and 0 copies of the *apolipoprotein E* (*APOE*) *ε4* allele, the major late-onset AD susceptibility gene, and in CN *presenilin 1* (*PSEN1*) mutation carriers and non-carriers from the world’s largest autosomal dominant early-onset AD kindred in Antioquia, Colombia; we developed several image-analysis techniques and a composite cognitive measure with improved power; and we estimated the sample sizes needed when using these endpoints.

We recently proposed the Alzheimer’s Prevention Initiative (API) to help in the effort to launch an era in AD prevention research. The API will include preclinical AD treatment/biomarker development trials in individuals at increased genetic risk for early-onset and late-onset AD, an extremely large Colombian early-onset AD Prevention Registry; and an extremely large North American Alzheimer’s Prevention Registry to support several preclinical AD trials. The API’s first trial will test the anti-amyloid immunization therapy crenezumab in CN PSEN1 mutation carriers using the best established brain imaging and cerebrospinal fluid measurements and our composite cognitive measure as the primary clinical endpoint. It is intended to test an anti-amyloid agent in the preclinical treatment of AD, help determine the extent to which a treatment’s biomarker effects predict a clinical outcome, provide a better test of the amyloid hypothesis, and provide raw data and biological samples to the research community. With support from the U.S. National Institutes of Health, philanthropic funds from the Banner Alzheimer’s Institute, and Genentech, it is also intended to help provide a new paradigm for research collaboration in clinical trials.

In this presentation, we will briefly introduce the crucial role of flourodeoxyglucose positron emission tomography (PET), amyloid PET, structural and functional MRI in the preclinical detection, tracking, and treatment of AD. We will present the novel image processing methods we have developed to help in this endeavor, as well as their implementation in the API’s preclinical treatment/biomarker development programs.

